# Complete genome sequence of Flavobacteriaceae sp. strain GF1, isolated from the phycosphere of a coral endosymbiotic dinoflagellate

**DOI:** 10.1128/mra.01184-24

**Published:** 2024-12-31

**Authors:** Toshiyuki Takagi, Kako Aoyama, Keisuke Motone

**Affiliations:** 1 Atmosphere and Ocean Research Institute, The University of Tokyo, Kashiwa, Japan; 2 Graduate School of Frontier Sciences, The University of Tokyo, Kashiwa, Japan; 3 Graduate School of Engineering, Osaka University 13013 , Suita, Japan; University of Southern California, Los Angeles, California, USA

**Keywords:** carotenoid-producing bacteria, coral, endosymbiotic dinoflagellate, phycosphere

## Abstract

A Flavobacteriaceae sp. strain GF1 was isolated from an endosymbiotic dinoflagellate of a coral, and the genome was sequenced using a PacBio Sequel IIe system. The genome consists of a circular 5,300,001 bp chromosome and is predicted to harbor 6 rRNA genes, 42 tRNA genes, and 4,465 coding sequences.

## ANNOUNCEMENT

Carotenoid-producing bacteria are widespread in coral reef ecosystems, including in coral hosts, their endosymbiotic dinoflagellates, macroalgae, and the surrounding seawater (1–7). Among them, a marine Flavobacteriaceae sp. strain GF1 was isolated from the phycosphere of an endosymbiotic dinoflagellate (family Symbiodiniaceae) of the coral, *Galaxea fascicularis* (3). Taxonomic identification using the EzBioCloud database (8) confirmed that the 16S rRNA gene sequence of strain GF1 is most closely related to *Muricauda lutaonensis* CC-HSB-11^T^ (95.7% similarity). GF1 protects the endosymbiotic dinoflagellate from environmental stress and inhibits ROS generation through the production of the carotenoid zeaxanthin (3). The genus *Muricauda* is reportedly one of the core members of bacterial communities in Symbiodiniaceae cultures (9). To better understand this strain’s genetic properties, we provide the complete genome sequence of Flavobacteriaceae sp. strain GF1.

A single colony of GF1 was purified in 1/10 strength ZoBell’s 2216E agar medium. GF1 was grown in marine broth (BD Biosciences, Franklin Lakes, NJ, USA) at 25°C for 36 h. Cultured cells were harvested by centrifugation, and genomic DNA was extracted using a Qiagen Genomic-tip 20 /G and Qiagen DNA Buffer Set (Qiagen, Hilden, Germany). After fragmenting the genomic DNA to 10–20 kb using g-TUBE (Covaris, Woburn, MA, USA), a SMRTbell Express Template Prep Kit v2.0 (Pacific Biosciences [PacBio], Menlo Park, CA, USA) was used for library preparation following the manufacturer’s recommendations. A SMRTBell polymerase complex was obtained using a binding kit 2.2 (PacBio) and sequenced with a PacBio Sequel IIe (PacBio) by Bioengineering Lab. Co., Ltd. (Kanagawa, Japan). The raw-read N50 is 13,330 bp. Default parameters were used for all software unless otherwise specified. Sequence data were imported into SMRT Link v.10.1.0.119528 to obtain high-fidelity (HiFi) reads with quality values > 20. Using Filtlong v.0.2.0 (https://github.com/rrwick/Filtlong), HiFi reads of >1,000  bp were selected, which yielded 65,119 reads (parameters: minimum length 1000 and keep percent 90). The resultant reads were used to assemble contigs with Flye v.2.8.3 (10), which yielded a single contig (5,300  kb in length, with 162-fold coverage).

CheckM v.1.2.2 was used to check the quality of the assembled genome sequence (11), with completeness of 99.0% and contamination of 2.9%. Genome annotation was performed using the DDBJ Fast Annotation and Submission Tool (DFAST) (https://dfast.ddbj.nig.ac.jp/) (12). The complete Flavobacteriaceae sp. strain GF1 genome comprises a circular 5,300,001 bp chromosome and exhibits a G + C content of 43.2%, with 4,465 coding sequences, six rRNA genes, and 42 tRNA genes.

## Data Availability

The assembled genome sequence and annotations for Flavobacteriaceae sp. strain GF1 were deposited in DDBJ/EMBL/GenBank under accession number AP035880, and the sequencing data were deposited in the Sequence Read Archive (SRA) under BioProject accession number PRJDB18432.
